# Temporally Associated Invasive Pneumococcal Disease and SARS-CoV-2 Infection, Alaska, USA, 2020–2021

**DOI:** 10.3201/eid2909.230080

**Published:** 2023-09

**Authors:** Katherine Newell, Marc Fischer, Stephanie Massey, Laurie Orell, Jonathan Steinberg, Megan Tompkins, Louisa Castrodale, Joseph McLaughlin

**Affiliations:** Centers for Disease Control and Prevention, Atlanta, Georgia, USA (K. Newell, M. Fisher, L. Orell, J. Steinberg);; Alaska Division of Public Health, Anchorage, Alaska, USA (K. Newell, S. Massey, M. Tompkins, L. Castrodale, J. McLaughlin)

**Keywords:** Streptococcus pneumoniae, bacteria, pneumococcal infections, SARS-CoV-2, COVID-19, respiratory infections, co-infection, temporally associated, invasive pneumococcal disease IPD, zoonoses, Alaska, United States

## Abstract

*Streptococcus pneumoniae* can co-infect persons who have viral respiratory tract infections. However, research on *S. pneumoniae* infections that are temporally associated with SARS-CoV-2 infections is limited. We described the epidemiology and clinical course of patients who had invasive pneumococcal disease (IPD) and temporally associated SARS-CoV-2 infections in Alaska, USA, during January 1, 2020–December 23, 2021. Of 271 patients who had laboratory-confirmed IPD, 55 (20%) had a positive SARS-CoV-2 test result. We observed no major differences in age, race, sex, or underlying medical conditions among IPD patients with and without SARS-CoV-2. However, a larger proportion of IPD patients with SARS-CoV-2 died (16%, n = 9) than for those with IPD alone (4%, n = 9) (p<0.01). IPD patients with SARS-CoV-2 were also more likely to be experiencing homelessness (adjusted OR 3.5; 95% CI 1.7–7.5). Our study highlights the risk for dual infection and ongoing benefits of pneumococcal and COVID-19 vaccination, especially among vulnerable populations.

Invasive pneumococcal disease (IPD) occurs when *Streptococcus pneumoniae* infects a normally sterile site, such as blood or cerebrospinal fluid. Viral respiratory tract infections caused by rhinovirus, respiratory syncytial virus, and influenza virus are known to predispose patients to secondary bacterial infections, including IPD (*1*,*2*). Bacterial infections in patients who have viral respiratory tract infections also have been associated with greater disease severity and increased mortality rates (*3*–*7*).

Despite the widespread global circulation of SARS-CoV-2, a limited number of studies have examined IPD and SARS-CoV-2 co-infections. A meta-analysis of case series reported that only 4% of patients who had COVID-19 had a bacterial co-infection and 14% had a bacterial secondary infection (*8*); no predominant bacterial pathogen was reported. Those bacterial infections occurred mainly among patients in intensive care units. Based on previous studies, only a small proportion of SARS-CoV-2 infections are accompanied by IPD (*9*,*10*); however, outcomes of such cases might be more severe (*11*). A recent national cohort study in England reported that, although substantial nationwide decreases were observed in IPD incidence during the COVID-19 pandemic, persons who had IPD and SARS-CoV-2 co-infections had higher case-fatality rates than did patients who had IPD alone, particularly among older adults (*12*).

In the United States, Alaska has consistently had among the highest IPD rates in recent years, and a disproportionate burden occurs among Alaska Native persons (*13*–*16*). However, the possible interactions between IPD and SARS-CoV-2 infections are unclear. We evaluated and compared the epidemiology and clinical course of patients with IPD with and without temporally associated SARS-CoV-2 infection in Alaska during 2020‒2021.

## Methods

### Data Sources

The Alaska Division of Public Health and the US Centers for Disease Control and Prevention Arctic Investigations Program maintain statewide laboratory-based surveillance for invasive disease caused by *S. pneumoniae*. Data are collected regarding patient demographic characteristics, clinical syndrome, pneumococcal vaccination, and illness outcomes through medical records. We included patients with IPD in Alaska during January 1, 2020–December 23, 2021. SARS-CoV-2 infection status was determined from nucleic acid amplification test and antigen test results reported to the Alaska Division of Public Health. COVID-19 testing procedures were based on State of Alaska COVID-19 guidance, including testing all symptomatic persons, as well as any asymptomatic person at admission to a healthcare facility. We linked cases by unique patient identifiers. We excluded patients with IPD who had no SARS-CoV-2 testing performed within 30 days before or after their positive *S. pneumoniae* culture. COVID-19 vaccination status was assigned through linkage with the immunization information system of Alaska.

### Definitions

We defined a case of IPD as *S. pneumoniae* isolated from or bacterial DNA detected in a normally sterile site, including blood, cerebrospinal fluid, pleural fluid, peritoneal fluid, pericardial fluid, joint fluid, bone, or deep tissue, in an Alaska resident. We defined temporally associated SARS-CoV-2 infection as a positive SARS-CoV-2 test result detected on a respiratory specimen collected within 30 days before or after the specimen collection date of the *S. pneumoniae* culture or positive DNA test result. We defined underlying medical conditions as those conditions specified at the time of IPD reporting that are established risk factors for IPD, including chronic lung disease, cardiovascular disease, immunosuppression, alcoholism, chronic renal disease, current smoking, or diabetes (*17*). We defined patients as being fully vaccinated against COVID-19 if they had received the second dose of the Pfizer-BioNTech (https://www.pfizer.com) or Moderna (https://www.modernatx.com) mRNA vaccines or 1 dose of the J&J/Janssen (https://www.jnj.com) vaccine >14 days before *S. pneumoniae* detection. We assigned COVID-19 vaccination status only to IPD patients who were eligible for COVID-19 vaccination at the time of IPD detection, based on the initial COVID-19 vaccine introduction schedule of Alaska. Pneumococcal vaccination was having received >1 dose of 13-valent pneumococcal conjugate vaccine (PCV13) or 23-valent pneumococcal polysaccharide vaccine (PPSV23) >14 days before *S. pneumoniae* detection. We defined IPD serotype groups as *S. pneumoniae* serotypes contained in PCV13 plus serotype 6C, those contained in PPSV23 but not in PCV13, and nonvaccine and unknown serotypes.

### Statistical Analysis

We compared demographic, epidemiologic, and clinical characteristics of IPD patients with and without a temporally associated SARS-CoV-2 infection. We used χ^2^ or Fisher exact tests for categorical variables. We performed multivariable logistic regression to identify risk factors for IPD and temporally associated SARS-CoV-2 infection among all patients with IPD. We included known risk factors associated with SARS-CoV-2 infection and retained those that resulted in the best fit models selected by the Akaike Information Criteria through backward stepwise selection. We used R version 4.1.1 (https://www.R-project.org) for all statistical analyses. This activity was reviewed by CDC and was conducted consistent with applicable federal law and CDC policy (see, e.g., 45 C.F.R. part 46.102(l)(2), 21 C.F.R. part 56; 42 U.S.C. §241(d); 5 U.S.C. §552a; 44 U.S.C. §3501 et seq.).

## Results

During January 2020–December 2021, we identified 330 IPD case-patients. Of those persons, 59 (18%) had no SARS-CoV-2 testing performed within 30 days before or after IPD specimen collection date and were thus excluded from the analysis. Of the excluded persons, 38 (64%) did not undergo SARS-CoV-2 testing because of having a positive culture for *S. pneumoniae* before COVID-19 testing was initiated in Alaska in March 2020. Other persons might have obtained a positive test result for COVID-19 within the previous 90 days; per testing guidance, these persons were exempted from further testing. 

Of the remaining 271 IPD case-patients, 55 (20%) had a temporally associated SARS-CoV-2 infection (Figure). Of those 55 patients, 49 (89%) had a positive SARS-CoV-2 test result on a specimen collected within 30 days before or on the same day as specimen collection for *S. pneumoniae* detection (Table 1). Only 6 patients had a positive SARS-CoV-2 test result on a specimen collected after their positive test for *S. pneumoniae.* All IPD patients who died and had a temporally associated SARS-CoV-2 infection (n = 9) had SARS-CoV-2 detected before or concurrent with their IPD; no deaths occurred among patients who had SARS-CoV-2 detected >24 hours after a positive test result for *S. pneumoniae*.

**Figure:**
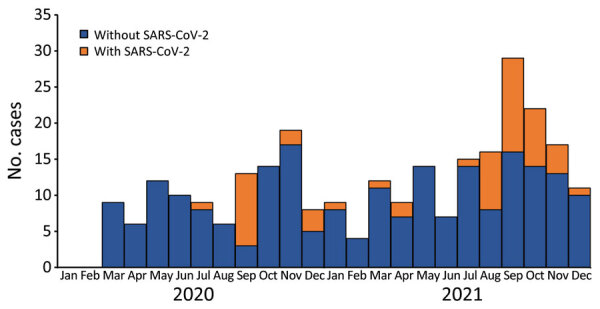
Epidemic curve for invasive pneumococcal disease case-patients with and without temporally associated SARS-CoV-2 infections, by month invasive pneumococcal disease specimen was collected, Alaska, USA, 2020–2021.

**Table 1:** Timing of SARS-CoV-2 detection in patients who had IPD and temporally associated SARS-CoV-2 infection, Alaska, USA, 2020–2021*

Timing of SARS-CoV-2 detection†	Nonfatal cases, no. (%), n = 46	Fatal cases, no. (%), n = 9
1‒30 d before IPD	21 (46)	6 (66)
Same day as IPD	19 (41)	3 (33)
1‒30 d after IPD	6 (13)	0

*IPD, invasive pneumococcal disease. †Difference between dates of specimen collection.

Seven (3%) IPD cases were reported among patients <20 years of age (Table 2), including 1 patient who had a temporally associated SARS-CoV-2 infection (detected 9 days before IPD). The remaining 264 (97%) patients who had IPD were adults >20 years of age (median age 52 years, range 20–88 years); 54 had a temporally associated SARS-CoV-2 infection. No major differences in sex, age, race, region, or underlying medical conditions were reported among IPD patients with and without temporally associated SARS-CoV-2 infection (Table 2). A total of 19 (35%) of 55 patients who had IPD and temporally associated SARS-CoV-2 infections were persons experiencing homelessness, compared with 39 (18%) of 216 patients with IPD alone (p = 0.01). IPD cases with temporally associated SARS-CoV-2 infection were more likely to occur during July‒September 2021, the period when COVID-19 hospitalizations peaked in Alaska (Table 2) (*18*).

**Table 2:** Characteristics of patients who had IPD temporally associated with a SARS-CoV-2 infection, compared with patients who had IPD alone, Alaska, USA, 2020–2021*

Characteristic	IPD patients with SARS-CoV-2 infection, no. (%), n = 55	IPD patients without SARS-CoV-2 infection, no. (%), n = 216	p value†
Sex			0.29
M	31 (56)	141 (65)	
F	24 (44)	75 (35)	
Age, y			0.90
<20	1 (2)	6 (3)	
20–39	12 (22)	57 (26)	
40–59	25 (46)	93 (43)	
≥60	17 (31)	60 (28)	
Race			0.98
Alaska Native/American Indian	29 (53)	117 (54)	
White	16 (30)	62 (29)	
Other/unknown	10 (18)	37 (17)	
Region			0.70
Anchorage	28 (51)	111 (51)	
Interior	7 (13)	31 (14)	
Gulf	4 (7)	16 (7)	
Matanuska-Susitna	5 (9)	15 (7)	
Northern	2 (4)	20 (9)	
Southeast	2 (4)	7(3)	
Southwest	7 (13)	16 (7)	
Underlying medical condition			1.0
≥1	44 (80)	175 (81)	
None	11 (20)	41 (19)	
Seasonality			<0.01
Summer, June–August	10 (18)	54 (25)	
Fall, September–December	37 (67)	76 (35)	
Winter, December–February	5 (9)	27 (13)	
Spring, March–May	3 (6)	59 (27)	
Person experiencing homelessness			0.01
Yes	19 (34)	39 (18)	
No	36 (66)	177 (82)	
Pneumococcal vaccine received			0.80
PCV13	5 (9)	26 (12)	
PPSV23	17 (31)	70 (32)	
Both	2 (4)	13 (6)	
Neither	31 (56)	107 (50)	
COVID-19 vaccine received			0.09
Fully vaccinated	9 (16)	43 (20)	
Not fully vaccinated	28 (51)	59 (27)	
Not eligible‡	18 (33)	114 (53)	
Clinical syndrome§			<0.01
Pneumonia	46 (84)	155 (72)	
Bacteremia without source	9 (16)	43 (20)	
Other	2 (4)	49 (23)	
Hospitalized			0.26
Yes	49 (89)	179 (83)	
No/unknown	6 (11)	37 (17)	
Died			<0.01
Yes	9 (16)	9 (4)	
No	46 (84)	196 (91)	
Unknown	0	11 (5)	

*IPD, invasive pneumococcal disease; PCV13, 13-valent pneumococcal conjugate vaccine; PPSV23, 23-valent pneumococcal polysaccharide vaccine. †By χ^2^ test. ‡Not eligible for COVID-19 vaccination at the time of IPD detection based on Alaska’s initial COVID-19 vaccine introduction schedule. Not included in χ2 calculation. §Clinical syndromes are not mutually exclusive.

Patients who had IPD and temporally associated SARS-CoV-2 infection were more likely to show a clinical syndrome of pneumonia defined in the patient’s medical records, but we found no differences in rates of hospitalization between patients with or without temporally associated SARS-CoV-2 infection. Among 55 IPD patients who had temporally associated SARS-CoV-2 infection, 9 (16%) died, compared with 9 (4%) of 216 patients who had IPD alone (p<0.01).

Of 271 patients who had IPD, 133 (49%) had received >1 dose of pneumococcal vaccine; no difference was reported in pneumococcal vaccination coverage rates between patients with or without temporally associated SARS-CoV-2 infection. Of 139 patients who had IPD and were eligible for COVID-19 vaccination, only 52 (37%) had completed a COVID-19 vaccine primary series. No difference in COVID-19 vaccination rates was reported between patients with or without temporally associated SARS-CoV-2 infection.

Overall, 30 (55%) of the IPD cases among patients who had SARS-CoV-2 infections were caused by *S. pneumoniae* serotypes contained in the PCV13 vaccine, compared with 167 (63%) of the IPD cases in patients without SARS-CoV-2 infection (Table 3). Serotype 4 was the most common cause of IPD among both groups, including 44% (24/55) of IPD cases with SARS-CoV-2 infection and 56% (120/216) of IPD cases without SARS-CoV-2. In contrast, a higher proportion of patients who had temporally associated SARS-CoV-2 infection had IPD attributable to a serotype contained in PPSV23 but not PCV13 or a serotype that is not in either vaccine. Multivariable analysis showed that, among patients who had IPD, persons experiencing homelessness were more likely to have a temporally associated SARS-CoV-2 infection than persons not experiencing homelessness (adjusted odds ratio 3.5, 95% CI 1.7–7.5) (Table 4).

**Table 3:** *Streptococcus pneumoniae* serotype by vaccine type among patients who had IPD temporally associated with SARS-CoV-2 infection or IPD alone, Alaska, USA, 2020–2021*

*S. pneumoniae* serotype by vaccine type	IPD patients with SARS-CoV-2 infection, no. (%), n = 55	IPD patients without SARS-CoV-2 infection, no. (%), n = 216
PCV13 + 6C†	30 (55)	137 (63)
PPSV23, non-PCV13‡	18 (33)	50 (23)
Nonvaccine type	6 (11)	9 (4)
Unknown	1 (2)	20 (9)

*IPD, invasive pneumococcal disease; PCV13, 13-valent pneumococcal conjugate vaccine; PPSV23, 23-valent pneumococcal polysaccharide vaccine. †Serotypes contained in PCV13 (i.e., 1, 3, 4, 5, 6A, 6B, 7F, 9V, 14, 18C, 19A, 19F, 23F) plus serotype 6C. ‡Serotypes contained in PPSV23 but not in PCV13 (i.e., 2, 8, 9N, 10A, 11A, 12F, 15B, 17F, 20, 22F, 33F).

**Table 4:** Multivariable analysis of risk factors for invasive pneumococcal disease temporally associated with a SARS-CoV-2 infection compared with patients who had with IPD alone, Alaska, USA, 2020–2021

Characteristic	Adjusted odds ratio* (95% CI)
Person experiencing homelessness	
No	Referent
Yes	3.0 (1.4‒6.7)
Sex	
M	Referent
F	1.8 (0.9‒3.6)
Age, y	
<50	Referent
≥50	2.0 (1.0‒3.9)
Race	
White	Referent
American Indian/Alaska Native	0.9 (0.4‒1.9)
Other/unknown	0.9 (0.3‒2.5)
Underlying medical condition	
None	Referent
One or more	0.8 (0.3‒1.8)
Season	
Summer, June–August	Referent
Fall, September–December	2.7 (1.2‒6.4)
Winter, December–February	0.9 (0.3‒3.1)
Spring, March–May	0.3 (0.1‒1.2)
Fully vaccinated for COVID-19	
Yes	Referent
No	2.0 (0.9‒4.1)

*Multivariable logistic regression model mutually adjusted for other variables.

## Discussion

We found that, among 271 patients who had laboratory confirmed IPD, 55 (20%) also had a temporally associated SARS-CoV-2 infection. For most of those patients, SARS-CoV-2 infection was detected before or concurrent with IPD. This finding is similar to what has been observed for other viral infections, such as influenza viruses, rhinoviruses, and adenoviruses (*19*–*22*). The mechanism through which SARS-CoV-2 infection might predispose a person to IPD is unclear. However, as for other viral infections, the cause is likely multifactorial, including epithelial damage, changes in airway function, upregulation and exposure of receptors, inhibited immune response, or enhancement of inflammation (*23*–*26*).

We found that IPD patients who died were more likely to have a temporally associated SARS-CoV-2 infection. All patients who died and had IPD and a temporally associated SARS-CoV-2 infection also had their infection detected either before or concurrently with the IPD. This finding suggests secondary bacterial infection as a possible complicating factor for death. All deceased patients with a SARS-CoV-2 infection had COVID-19 listed as a cause of death. However, because of a lack of detailed clinical data and similar clinical manifestations of both diseases, we were unable to determine how IPD, SARS-CoV-2 infection, or a combination of both contributed to the patients’ deaths.

Among all reported patients who had IPD, persons experiencing homelessness were more likely to have a temporally associated SARS-CoV-2 infection. People experiencing homelessness are known to be at increased risk for IPD, probably attributable to staying in congregate settings, limited uptake of routine vaccinations, and higher prevalence of predisposing medical conditions (*27*–*31*). However, the possibility also exists that our findings were caused by increased detection of SARS-CoV-2 infection from routine screening in homeless shelters.

The first limitation of our study was that we were not able to obtain symptom onset dates for IPD or SARS-CoV-2 infection, indicating that timing of infection might differ from timing of detection in our results. Second, we were unable to determine how many SARS-CoV-2 tests persons experiencing homelessness received during the study period relative to the general population. Therefore, we could not calculate whether increased SARS-CoV-2 testing contributed to the observed increased odds of SARS-CoV-2 infection in persons experiencing homelessness. Third, our study was limited in assessing the interaction between homelessness and death because of the small number of deaths among persons experiencing homelessness. However, it is useful to recognize that homelessness itself has consistently been associated as an independent risk factor for increased deaths. Fourth, we were unable to obtain detailed clinical information regarding those patients who died and had IPD and a temporally associated SARS-CoV-2 infection, which indicates that we could not determine the etiologic role that SARS-CoV-2 had in their death. We also assigned COVID-19 vaccination eligibility based on the phased rollout in the Alaska general population. Certain IPD patients might have been eligible before this date on the basis of immunocompromising conditions and occupational risk factors (e.g., healthcare workers) and underestimated the number of eligible persons not vaccinated. We also did not examine risk factors for IPD and deaths because the total number of patients who died was small (n = 18) probably resulting in small sample bias from maximum-likelihood estimation in multivariable models. Fifth, because we only included patients who had IPD, we cannot infer the association concerning other noninvasive pneumococcal infections with SARS-CoV-2. Nevertheless, the study has multiple strengths, including linkage of statewide data sources to effectively capture all reported cases of IPD and COVID-19 in the state during the study period and a robust epidemiologic comparison of persons co-infected with IPD and SARS-CoV-2 to those infected with IPD alone.

In conclusion, we found that ≈1 of 5 patients who had IPD in Alaska during 2020–2021 had a temporally associated SARS-CoV-2 infection, and a greater proportion of patients who had IPD and temporally associated SARS-CoV-2 infection died compared with persons who had IPD alone. Persons experiencing homelessness who had IPD were at increased risk for temporally associated SARS-CoV-2 infection. Healthcare providers should be aware of the added risks associated with dual infection and the ongoing benefits of pneumococcal and COVID-19 vaccination, especially among vulnerable populations (*17**,**32*,*33*).
